# Biomimetics with Trade-Offs

**DOI:** 10.3390/biomimetics8020265

**Published:** 2023-06-17

**Authors:** Julian Vincent

**Affiliations:** School of Engineering and Physical Science, Heriot-Watt University, Edinburgh EH14 4AS, UK; j.vincent@hw.ac.uk

**Keywords:** trade-off, dialectic, TRIZ, inventive principle, wood wasp ovipositor, intracranial endoscope, Pareto curve

## Abstract

Our knowledge of physics and chemistry is relatively well defined. Results from that knowledge are predictable as, largely, are those of their technical offspring such as electrical, chemical, mechanical and civil engineering. By contrast, biology is relatively unconstrained and unpredictable. A factor common to all areas is the trade-off, which provides a means of defining and quantifying a problem and, ideally, its solution. In order to understand the anatomy of the trade-off and how to handle it, its development (as the dialectic) is tracked from Hegel and Marx to its implementation as dialectical materialism in Russian philosophy and TRIZ, the Theory of Invention. With the ready availability of mathematical techniques, such as multi-objective analysis and the Pareto set, the trade-off is well-adapted to bridging the gaps between the quantified and the unquantifiable, allowing modelling and the transfer of concepts by analogy. It is thus an ideal tool for biomimetics. An intracranial endoscope can be derived with little change from the egg-laying tube of a wood wasp. More complex transfers become available as the technique is developed. Most important, as more trade-offs are analyzed, their results are stored to be used again in the solution of problems. There is no other system in biomimetics which can do this.

## 1. Introduction

For more than 100 years, biology has been the recipient of the wisdom of engineers, physicists and chemists, and the time has come to return the favor. However, there is a problem. Engineering is numerate, and biology is largely descriptive.

Biological systems, understood at the level of the phenotype, are not in equilibrium with their environment, not reversible in their action or evolution and are open—they interact with their unpredictable and often chaotic surroundings. Further, these are all characteristics that physics finds difficult or impossible to cope with. Whilst physics (and physics-as-chemistry) can produce a believable model of biological phenomena at the base level of the genotype, it starts to fail if we move to the production of proteins and become enmeshed with the phenotype. At this level, physics can provide only a restricted view of biology. Although the analysis provided by physics can be applied to isolated components of living matter and we can learn much about individual processes and materials, the isolation of these processes cannot (yet) be reversed to recreate the whole; we cannot reintegrate them. Biology and physics are incompatible, at least in terms of the current ability of physics to model life. A successful model can quantify and predict. There is currently no way that biological events such as speciation and morphological change can be predicted from first principles. At its root, the current understanding of biology is unquantifiable in terms of constitutive models. It is comparative.

A possible solution may be found in the thinking of ancient Greeks in the person of Socrates. His methods of rational discussion made his interlocutors aware that their moral theories or philosophical doctrines were often inconsistent and therefore untenable. His way of thinking was to consider opposing opinions and approaches and to resolve them. This approximates to the dialectic. Over the years, many forms of dialectic have arisen; the one most familiar to Europeans is due to the German philosopher Hegel. He posed a concept—the thesis—and negated it with the antithesis. The resolution—the negation of the negation—is the synthesis which can then become the thesis in a new phase of the dialectic. This is the simplest form of the dialectic. As a logical construct this is acceptable, but it cannot be scientific because it is not based on demonstrable and repeated results—empiricism. It is, however, a useful concept for defining what is known. This is comforting for biologists because trial and error is the basis of natural selection, genetic and phenotypic variations being exposed to the selection pressures of the environment, physical and biotic. However, the trial and error of natural selection, whose eventual product is evolution, is different from the Socratic and Hegelian versions of the dialectic in at least one major factor. Socrates could argue only about what was known, and Hegel’s formalism is even more limiting.

Applied to biology, the adaptive changes that organisms undergo relative to whatever may be the current state of affairs are essentially unpredictable, because they emerge epigenetically as products of intrinsic genetics and an extrinsic and unpredictable context. Natural selection then works on variants of organisms that have some novelty about them; the selection pressures are similarly lacking in control, although they may be circumscribed by context. One should remember, though, that the initiation of a “trial” or test will nearly always carry some memory of previous trials, variously as experience, morphology, physiology or questing intelligence of enquirer and organism alike. “Trial and error” is not a process of random choice but neither is it as tightly corseted as the dialectic approach. Randomness will always allow a larger part of the solution space to be sampled; this is advantageous for survival. Research is much the same—you may have an inkling of what the answer has to be but the journey you take to get there is unlikely to be direct. It is also likely that your imagined end point will turn out to have been illusory, and the new reality is more interesting and convincing than was initially conceived. In science, therefore, and especially in biology (biologists love surprises), the dialectic is not an appropriate model for research because at least half of the argument cannot be predicted because there is no coherent model that will support prediction.

In fact, it would be best not to use the term “dialectic” in scientific discourse. However, although the experimental approach to a pragmatic understanding of our world is best achieved by observation and experiment, the statistics and complexity of everyday interactions show that arrival at the present state almost always involves a trade-off.

## 2. The Trade-Off and TRIZ

The system described in this paper is based on TRIZ (a Russian acronym, properly translated as the theory of solving problems inventively). TRIZ was chosen because it has its roots in engineering, and the ultimate target of this project was to solve problems for engineers. Some history is useful to explain the origins of TRIZ and the modifications I have made.

Hegel collaborated with Karl Marx, another well-known philosopher. They developed dialectic materialism, a concept that is confrontational, requiring any problem to be expressed as the tension between the two questions “what do I want?” and “what is stopping me getting there?”. The simple resolution of this form of dialectic is elimination of the obstacle, though it is often not that clear cut. Note that it is very similar to the “negation of the negation” described above.

TRIZ, based on dialectic materialism by the engineer Genrich Altshuller, is a development of this feature of Russian thinking that is included in the Russian school curriculum. Its various techniques, some common to other problem-solving systems, have as their basis a two-dimensional matrix in which the two halves of the dialectic—thesis and antithesis—are arranged along the two sides of a matrix, leaving the body of the matrix to contain clues to the synthesis of the dialectic so defined. Thesis and antithesis become the definition of the problem being investigated. They were renamed Parameters and the problem any two of them define is the Contradiction—hence the Contradiction Matrix of TRIZ. Some confusion can enter at this point because the Parameters each can make a positive or negative contribution to the dialectic, which means that the two sides of the matrix are populated by the same list of words. The convention is that the horizontally arranged list is the antithesis—the characteristic that is getting worse—and the vertical axis is the thesis—the characteristic that is improving. Thus, the two sides of the matrix represent different aspects of the dialectic. The synthesis became the Inventive Principle defining what needs to be changed to achieve a solution to the Contradiction, indexed within the matrix. Conceptually, the Inventive Principles should be different depending on whether a particular Parameter defines a worsening or improving feature—i.e., is the thesis or the antithesis. The Contradiction Matrix is therefore a full square. In classical TRIZ, there are 39 Parameters and 40 Inventive Principles, each identified by a serial number. Analysis of many “strong” patents showing great originality provided data for the Matrix. As far as I know, there is no record of which patents were examined nor the data those patents contributed, nor has the quality of the Parameters or Inventive Principles been subjected to statistical analysis of any sort, either to test their completeness of coverage of the problem space in engineering or any degree of overlap they might have which would affect their accuracy or applicability as descriptors. It may be only in the age of computing and network theory that such questions might be formulated or answered.

Despite the polarization of the Parameters, simple analysis shows that half the Contradiction Matrix is symmetrical about its diagonal. The Matrix can therefore be generalized as a classification of unpolarized trade-offs, for which there is much mathematical support and theory—Pareto modelling, multi-objective analysis, etc. In the context of dialectical materialism, the trade-off is considered a weak formalization. Hegel and Marx would not be pleased.

## 3. The Strength of the Trade-Off

Trade-offs such as speed–accuracy, cost–benefit and stability–adaptability are high-level abstractions that can define problems in all disciplines, not just engineering but also biology, management, food science, architecture, medicine, behavior, etc. Their power is in analogy and pattern [1,2]. The implication is then that the Parameters that define the trade-off (referred to here as Trade-Off Parameters, or TOPs) and the Inventive Principles (IPs) that define the changes that will allow the current system to accommodate or overcome the trade-off are of universal significance. Because trade-offs can bridge between disciplines, it is important that the TOPs and IPs should represent a moderately even and complete coverage of all possible trade-offs and their solutions (i.e., changes or adjustments) that resolve trade-offs. Necessarily, in their original TRIZ form, they are terse and require a degree of (or in?) interpretation and are obviously tuned to engineering. I have been developing the TRIZ Matrix to cope with inputs and outputs from both biology and engineering, with a terse lexicon exemplifying and amplifying the meaning and interpretation of the TOPs and IPs, derived from analysis of the words used to describe them in scientific reports of analyzed trade-offs.

## 4. Indexing and Accessing Trade-Offs

I have incorporated these developments into an ontology [3], an adaptable vehicle for the establishment and classification of a network of objects and actions. Because an ontology arranges things, ideas and processes (“entities”) in hierarchies, it can support the lexical expansion of each TOP and IP. In a standard TRIZ text, there are rarely more than four or five examples given to expand any one of the TOPs or IPs. My expansion of this system has been from the standpoint of a domain expert rather than resorting to any rigorous analysis. It is more important to answer the question “does it work?” before trying to fine-tune the system. Accordingly, each TOP and IP has been expanded in a branching hierarchy which is diagrammed in the figures in this paper. Within the ontology editor—Protégé—any term can be searched for, speeding up the assignment of a TOP to the definition of the trade-off in the initial stages of analysis, and similarly for the assignment of an IP to the resolution of the trade-off. This means that there can be two levels of definition of any IP or TOP: the more precise one represented by a leaf in the tree structure of the hierarchy and the generalized one represented by the root which defines the main topic. In the database that provides the cases that inform the ontology, the relevant TOPs and IPs are represented in both ways. When a search is performed on the ontology, it is possible to use either as the search object. The result of the search is delivered as the root. Rough calculation suggests that it is possible for the ontology datastore to have perhaps 100,000 cases, all differently defined, and the search system can cope with some 750 different trade-offs—far more than probably exist. The system has plenty of spare capacity for differentiation.

The mechanics of all this as an ontology have been reported before [3] and they remain substantially the same. Importantly, the ontology is a repository of information bringing together all the elements of the trade-off and a datastore in which results are retained. Thus, rather than a database, which is essentially static, the ontology not only retains the information but integrates it with what is already in the datastore and can be interrogated in detail. The ontology can also be updated and so accommodate new information and modified or new methods of analysis.

## 5. The Trade-Off in Practice

**Figure 1:**
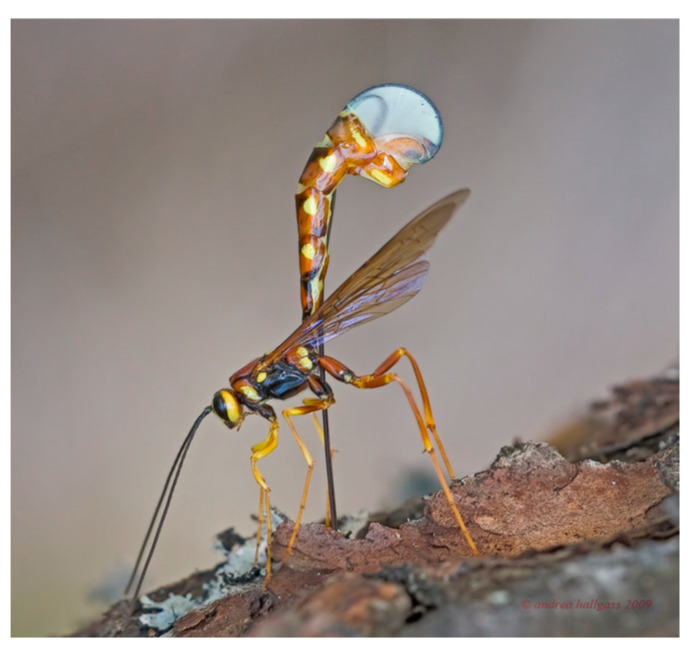
*Megarhyssa* drilling into wood with its ovipositor.

**Figure 2:**
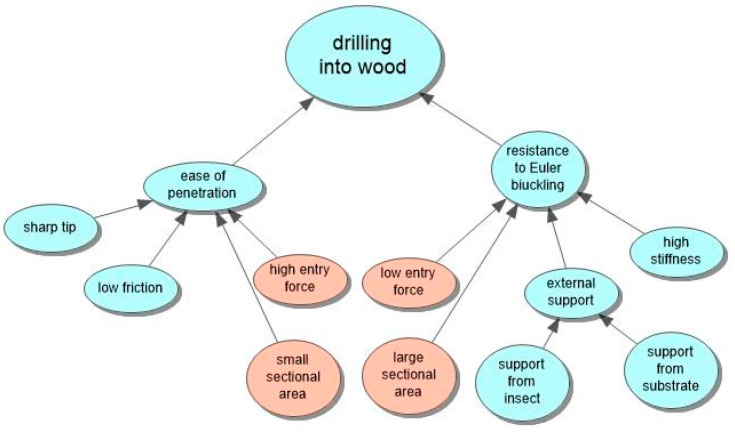
Factors leading to the apparent impasse. The orange bubbles define the basic conflict.

**Figure 3:**
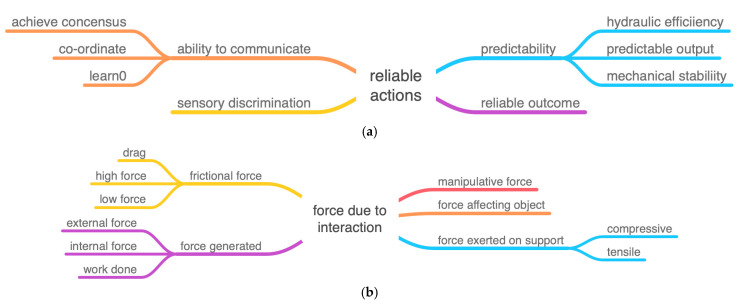
(**a**) TOP for the wasp ovipositor: reliable actions with the subset mechanical stability. (**b**) TOP for the wasp ovipositor: force due to interaction with the subset force generated.

The ichneumonoid wasp, *Megarhyssa nortoni* (Figure 1), parasitizes larvae of siricid wood wasps using its long thin ovipositor to drill into a rotting tree and cast around until it detects its prey deep within the wood. However, the ovipositor is too long and thin to be stable if it were simply pushed into the wood as one might with a twist drill or chisel—it would buckle. The wasp resists this in a number of ways. It keeps the free length of the drill as short as possible with clips that hold the drill into a groove under the abdomen, and it locates the drill between the bases of the hind legs where it emerges from the groove. Additional support is given by a sheath around the ovipositor that is pushed out of the way as the drill enters the wood. The drill is also very stiff. There still has to be a trade-off (Figure 2) between buckling and ease of penetration of the ovipositor [4,5]. The two components of this trade-off are represented by the TOPs “force due to interaction” and “reliable actions” (Figure 3a,b).

**Figure 4:**
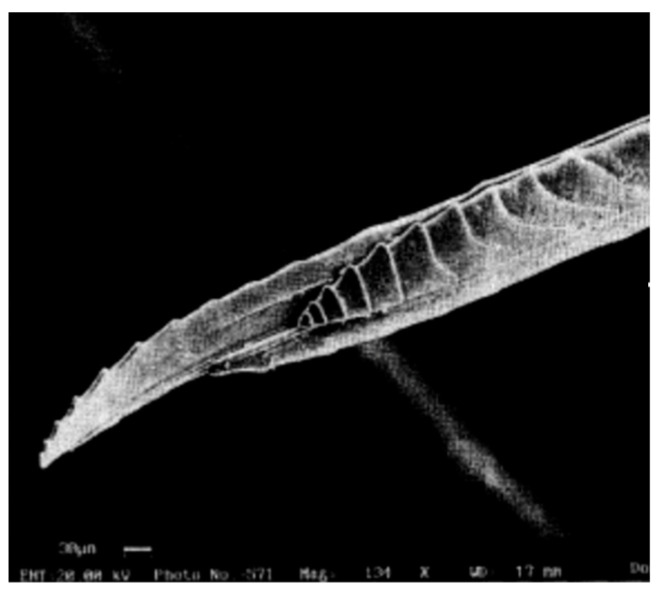
Ovipositor tip showing the reversed teeth and the prestrained end bending over.

**Figure 5:**
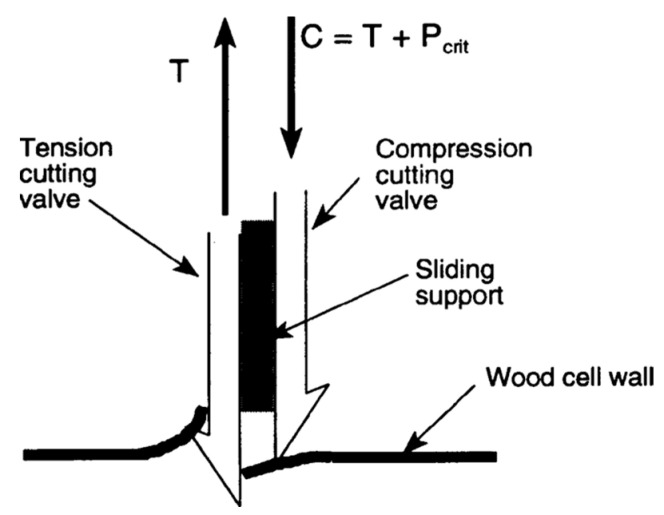
Forces at the tip of the ovipositor boring into a cell wall. T—tension; C—compression; P_crit_—critical Euler buckling load.

The solution is that the ovipositor avoids buckling by using reversed teeth at the tip (Figure 4) and linking the three parts together by sliding joints rather like the “zip-lock” mechanism on a plastic bag. Two parts can be anchored in the side of the hole and support a tensile force generated by the wasp. This tensile force stabilizes the other part of the ovipositor which is still free to slide up and down and can now be pushed further into the wood (Figure 5). Thus, external force is minimized and the wasp can provide all the force required despite having very little mass external to the drill. The factors involved in this resolution of the trade-off are shown in the following diagrams by standard IPs but with added lexical attributes. The diagrams show how these attributes are integrated within the hierarchy of each IP.

The diameter of the ovipositor is about 1 mm.

In its structure, the ovipositor uses the following characteristics:

**Figure 6:**
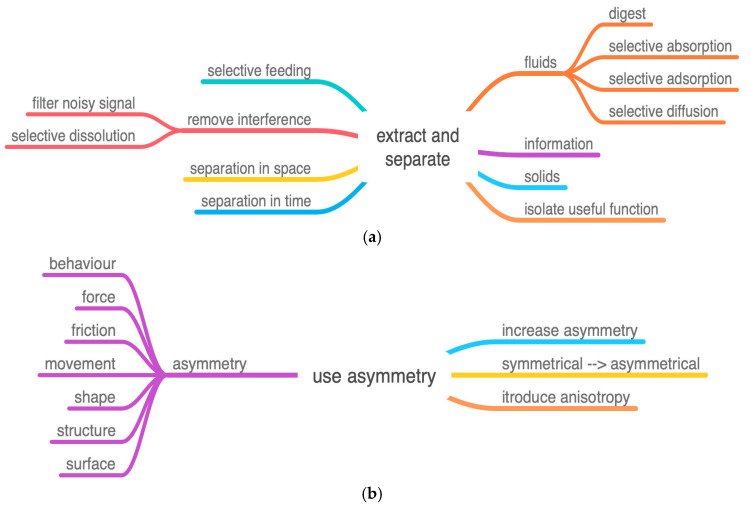

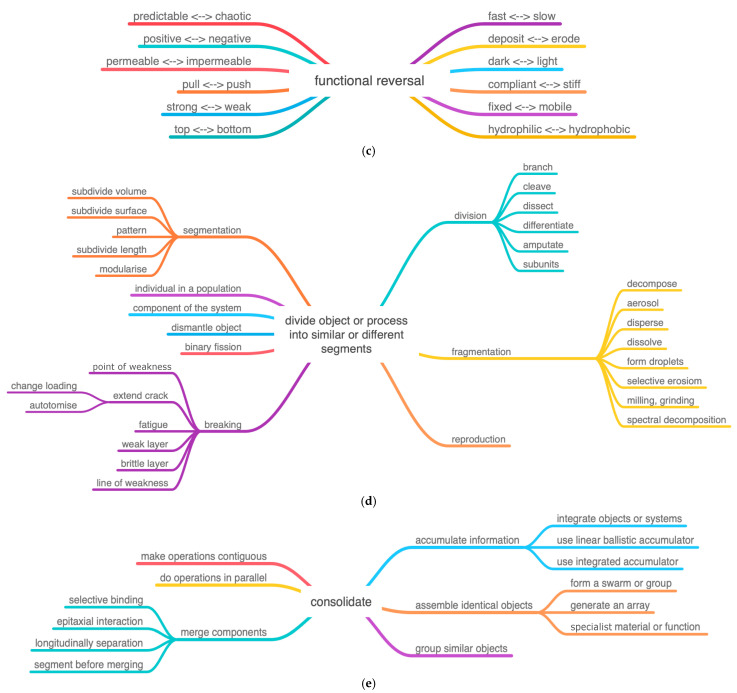
(**a**) IP Extract and separate. The remote actuation of the drill tip is a separation in space. (**b**) IP Use asymmetry. Includes both asymmetry of friction and asymmetry of shape. (**c**) IP Functional reversal. Alternate between pull and push. (**d**) IP Divide object or process into similar or different segments, subdivide length. (**e**) IP Consolidate. Two factors in the class merge components: longitudinal separation and segment before merging.

The choice of the TOPs and IPs is based on a paper [4] that, from mechanical tests, microscopy and search of the literature, estimated the severity of the problem of the ovipositor buckling during use and used the morphology of the ovipositor to deduce the combination of characteristics that solve the problem for the wasp. The topic was covered more completely by van Leeuwen and associates who confirmed the original observations and extended understanding of the design features [6]. The mechanism is found in the ovipositors of two groups of wasps (Siricidae and Ichneumonidae) and in the mouthparts of mosquitoes and certain Hemiptera-Homoptera.

## 6. A Biomimetic Product

These IPs (Figure 6), taken together, led to the design and production of a long, steerable, intracranial probe based on the solutions to the trade-off outlined above (https://www.youtube.com/watch?v=_wvAyiiuozs&t=63s, accessed on 14.06.2023). Ferdinando Rodriguez y Baena of the Medical Robotics department at Imperial College wanted to implement a steerable endoscope that could invade the brain but avoid damaging critical structures, blood vessels, etc. He could not see how he could implement such a device because he was unable to resolve the trade-off (Figure 2) until he was aware of the wood wasp ovipositor, which provided an answer. Several principles were missing from the wood wasp analysis or had to be reversed. The ovipositor is very stiff, whereas the brain endoscope is made of very soft plastic to be able to react against the soft tissue of the brain and bend around any important parts of the brain, blood vessels, etc. Vincent and King did not realize that the probe must match the stiffness of the substrate being entered. If they had compared the wood wasp with related wasps that drill into soft plants, they might have found that the stiffness of the probe is less, so compliance matching becomes more obvious. This would be available as a function of feedback but is not an obvious requirement until the mechanism of the ovipositor has been observed working in a variety of substrates. Another problem in implementing the design was that with the probe made of soft plastic: how well can the zip-lock mechanism between the moving segments maintain the joined integrity of the probe and not come apart under eccentric loading? This was treated as an engineering problem using multi-objective analysis to establish an optimum [7].

To date, the forces exerted by the wasp have not been measured and are conjectural based on published data describing the behavior of the wasp, the morphology of the ovipositor and some simple measurements of the buckling loads. Forces and the dynamics of the endoscope have been measured and found to be more complex. The wasp has been reported to drive the ovipositor in a rhythmic way; an experiment with the endoscope shows that the most effective strategy is to withdraw the endoscope slightly between thrusts and that this increases the rate of penetration [8].

The sensory organs available in some wasp ovipositors, presumably used to find a prey moth larva burrowing in the wood, have been replaced by a combination of X-ray mapping of the brain being probed that enables the calculation of a controlled trajectory for the probe.

## 7. Discussion

The example chosen to illustrate the use of trade-offs as a bridge between disciplines is as simple and obvious as it gets. The concepts can be transferred in literal form—there is little need for analogy. However, the IPs revealed by analyzing the wasp ovipositor as a trade-off have already proved useful in a related project—the mechanics of the sawfly’s saw. Sawflies cut into plant tissues to lay their eggs. They are closely related to horntail wasps, less closely to ichneumonoids such as *Megarhyssa*. However, they all pull the elements of saw or drill rather than push.

Trade-offs are better tested by their talent to generalize without a loss of detail. This is illustrated by eight examples, ranging from learning to play the piano to how a toad sees in low light, resolving the speed–accuracy trade-off with only four IPs [4]. In each instance, the roots of the Parameters were the same, but the details—the leaves—were different. Because an ontology is an “open world” construct, adjustments can be made to such details as more case studies are made available.

Trade-offs have a wider importance in biology and biomimetics than simply providing a framework for the universal definition and solution of a problem. For instance, it has been a long-held belief that the mechanisms and processes in organisms function at a level that is “just good enough” for survival, but not perfect [9]. The patterns of data that trade-offs reveal destroy this belief, for which there is no proof anyway, only the inability to live with the complexity of biology.

The “just good enough” hypothesis seems to be based on a suite of ideas such as

- that perfection takes more energy (though it may be more effective)
- that there are mechanisms that seem wasteful (though they may reduce risk)
- that perfection does not fit with the extent of variation observed within a species.

This approach can be refuted by realizing that any organism is a panoply of trade-offs resolved through evolution and that most trade-offs will have more than two TOPs in control—no organism can be optimal in all tasks at once [10]. “Just good enough” then changes from being a statement about the imperfection of nature to the realization that organisms, groups of organisms, species and communities are all systems. The “just good enough” hypothesis can apply only to an individual item within a system. Because every cell is a system, the hypothesis is irrelevant.

In its place, we have a network of trade-offs. A large part of the skill in biology is selecting an experimental system that isolates a single, simple trade-off [11]. Any graph of a simple trade-off will have a distribution of points bounded by a limit representing the maximum performance of the trade-off at any value [12]. This curve—the Pareto curve—maps out the best response of the trade-off. A clear example: a transport system that connects randomly distributed origins to a single target. In one half of the trade-off—“satellite”—each origin has a direct route to the target. This minimizes the distance between each origin and the target but maximizes the total of all the routes. The other extreme—“Steiner”—plots a route that connects many of the origins before arriving at the target. The total length of routes is minimal but the distance between an origin and the target will be longer for most of the points. These two are extremes of the trade-off of cost (maximum for satellite) and performance (maximum for Steiner). This is a universal trade-off that applies to road systems [12], architecture of plants and leaf-to-stem transport in plant vascular systems [13]. By varying the balance between cost and performance, a Pareto curve is generated representing the best outcome at any chosen balance between the two halves of the trade-off. In a series of experiments mapping the vascular system of tomato plants, Conn et al. [13] showed that the plants were achieving the best balance between cost and performance. Growing the plants under non-ideal conditions (e.g., low light intensity) changed the growth pattern of the plants, and the balance had moved along the curve. In other words, the plant was still performing as well as theory could predict given the circumstance. Thus, trade-offs do not simply define the problem; they can be used to define the best outcome under varying conditions, and in doing so demonstrate the adaptability of biological systems.

Ideally this example of a very common trade-off would have been analyzed so that the IPs could be invoked at the design stage of the network. The best attempt at this [14] maps the morphology of venation in a plant leaf, for which it produces believable results but has no underlying parameters other than relatively local responses to conditions that change as the network increases in size and complexity. By comparison, the venation of a real leaf is laid down in the early stages of development, apparently in the absence of such cues.

The trick is to tease some familiar order from the complexity and incipient chaos of life—in the limit this requires the conversion of unknown unknowns into known knowns. This is remarkably like Snowden’s Cynefin (a Welsh word pronounced “kun-ehvin”) framework, of which he says “Paradox and dialectical reasoning are key tools … in the un-ordered domains” [15]. Although biology is not an unordered domain, it clearly represents as such to those unused to its complexities, and many biomimicry practitioners talk of the millions of solutions to problems that organisms have solved in 3.8 billion years. This is clearly a total fiction and distinctly unhelpful. Organisms of all sorts are a collection of a finite number of systems (movement, sensing, digestion, etc.) identifiable from any textbook of comparative physiology. Heredity of the genetic code ensures that the basic mechanisms that drive these systems are consistent over swathes of species; adaptation is achieved by trade-offs within and between those systems in response to physical and biotic influences. It is not particularly easy to identify and unravel these trade-offs but they are there, real and rational, and they will submit to mathematical analysis [16]. The extraction and refinement of the trade-off strips away much of the biology, leaving a kernel of general truth and utility, transforming the bridge into an interface.

## Data Availability

The data was created with the aid of an ontology that has been under development for the last eight years. It is available on application to the author.
